# Editorial: Fertility preservation and restoration in oncologic and non-oncologic patients

**DOI:** 10.3389/fendo.2026.1988389

**Published:** 2026-09-21

**Authors:** Mahmoud Salama, Yasmin Jayasinghe, Mindy Christianson

**Affiliations:** 1 Department of Obstetrics, Gynecology and Reproductive Biology, Michigan State University, East Lansing, MI, United States; 2 Department of Obstetrics and Gynecology, University of Melbourne, Royal Women’s Hospital, Parkville, VIC, Australia; 3 Oncofertility Program, Royal Children’s Hospital, Melbourne, VIC, Australia; 4 Murdoch Children’s Research Institute, Melbourne, VIC, Australia; 5 Cleveland Clinic Fertility Center, Gynecology and Reproductive Biology Department, Cleveland Clinic Lerner College of Medicine, Cleveland, OH, United States

**Keywords:** cancer, fertility, non-oncologic patients, oncofertility, preservation, restoration

This Research Topic delineates the scope of fertility preservation as an essential component of multidisciplinary care for patients facing potential fertility loss due to gonadectomy or gonadotoxic treatments. The included articles highlight both established and emerging options for fertility preservation and restoration in oncologic and non-oncologic patients. Additionally, they emphasize the need to refine clinical guidelines, establish long-term outcome tracking through standardized registries, and implement equitable patient navigation structures and specialized oncofertility programs, particularly in resource-limited settings.

Ali et al. investigated the knowledge, practice, attitudes, perceptions and barriers regarding pediatric fertility preservation in resource-limited settings in Malaysia. This study surveyed 102 Malaysian healthcare providers (HCPs) using a validated 51-item questionnaire. Although a high proportion of HCPs demonstrated adequate knowledge (72%) and reported active patients’ referrals (>80%), specific discussion engagements remained low (17%), and a large majority exhibited negative overall attitudes (88.2%). Key obstacles included financial constraints for patients (30.4%), a lack of age-appropriate educational material (17.6%), and insufficient time for patient counseling prior to cancer treatments (12.7%). These findings underscore a critical need for policy support, public awareness initiatives, and specialized oncofertility programs.

Castellano et al. investigated the rate of testicular histology failure in predicting successful testicular sperm extraction (TESE). This retrospective study of 526 azoospermic patients evaluated the diagnostic concordance between traditional testicular histology examination by a histopathologist and immediate TESE sample examination by an embryologist, who examined the tubules for sperm cryopreservation. Concordance between the two methods was found in 70,7% of patients; whereas discordance was found in 29,3%. Among discordant cases, approximately 95% yielded viable spermatozoa via TESE that were absent or underestimated in the parallel testicular histology report. Consequently, relying solely on testicular histology risks false-negative predictions. To improve diagnostic accuracy, the authors recommend obtaining larger testicular biopsy for histological examination and using TESE cell suspension for cytological evaluation of spermatogenesis.

Weibring et al. investigated parenthood outcomes in a Swedish prospective cohort (1988-2020) of 1,378 adolescents and young adults (AYA) banking semen for fertility preservation at time of cancer diagnosis. In total, 493 men became fathers following cancer treatment: (81%) did so naturally, (18%) via assisted reproductive techniques including sperm cryopreservation, and (1%) through adoption. In this cohort, findings demonstrated a high spontaneous conception rate, and a relatively low utilization rate of cryopreserved sperm. Nevertheless, the authors highlighted that sperm cryopreservation remains a safe and reliable option for male fertility preservation particularly for patients at higher risk of fertility loss due to gonadectomy or gonadotoxic cancer treatments.

Gonen analyzed behavioral and economic factors influencing social oocyte cryopreservation decisions. This study applied conjoint analysis across a cohort of women aged 18–65 to identify the determinants affecting social egg freezing. Key predictors included baseline personal income, perceived risk of impending age-related infertility, and expected pregnancy success rates post-thaw. Findings highlight that while women view social egg freezing as a viable mechanism for reproductive autonomy, financial barriers dictate utilization. To improve access to social egg freezing, the author suggests updating the public health policies and expanding governmental financial support.

Wang et al. assessed the molecular and morphological dynamics during the 7-day 3D Matrigel *in vitro* culture of cryopreserved human ovarian tissue. DNA and RNA analyses after *in vitro* culture revealed a rise in gene fusion and alternative splicing events, potentially affecting gene expression and cellular functions. The authors concluded that long-time *in vitro* culture of cryopreserved human ovarian tissue results in substantial changes in its morphology and genetic alteration.

Sun et al. investigated the influence of ovarian cyst type and size on ovarian reserve markers with implications for fertility counseling and preservation strategies. In this retrospective cohort study of 474 reproductive-aged women, the presence of ovarian cysts was significantly correlated with reduced AMH and AFC, along with elevated FSH compared to the healthy control group. Both ovarian cyst type and size significantly influenced ovarian reserve, with endometriomas and large cysts demonstrating the most detrimental impact. In such cases, early ovarian reserve testing and personalized fertility preservation strategies are recommended.

Li et al. reported the diagnosis and management of a rare case of bilateral malignant mixed ovarian germ cell tumors (MOGCTs) in a 22-year-old female patient. Given the patient’s young age and her desire for future fertility, fertility-sparing surgery was performed to remove the tumors alongside surgical staging, followed by chemotherapy. At 12-month follow-up, the patient remained disease-free with regular menstruation and no signs of recurrence.

Turchinets et al. investigated the association between X chromosome aneuploidy in ovarian somatic cells and peripheral blood lymphocytes and ovarian function in adolescents with mosaic Turner syndrome. Five adolescents with mosaic Turner syndrome underwent assessment of ovarian reserve markers and ovarian tissue cryopreservation. Despite ongoing puberty and normal gonadotropin levels, reduced serum anti-Müllerian hormone (AMH) and inhibin B concentrations were observed in some participants. A normal X chromosome complement was identified in all analyzed oocytes, whereas ovarian stromal cells were predominantly monosomic for the X chromosome; in contrast, granulosa cells (GCs), demonstrated substantial intra- and inter-individual variability in X chromosome mosaicism. Furthermore, peripheral blood karyotype did not reliably reflect ovarian tissue mosaicism in this exploratory series. Therefore, a comprehensive evaluation of ovarian reserve markers is crucial when counseling patients with mosaic Turner syndrome regarding fertility preservation.

Brunette et al. outlined various processing methods for harvesting and testing ovarian tissue before and after cryopreservation. **Figure 1** provides a procedural overview of ovarian tissue collection, depicting the steps as performed at the National Institutes of Health (NIH), along with four research processing approaches (A-D) and their compatible experiment methodologies. The authors highlighted that as analysis options expand and research using ovarian tissue grows, it is crucial that tissue be properly processed and cryopreserved.

**Figure 1:**
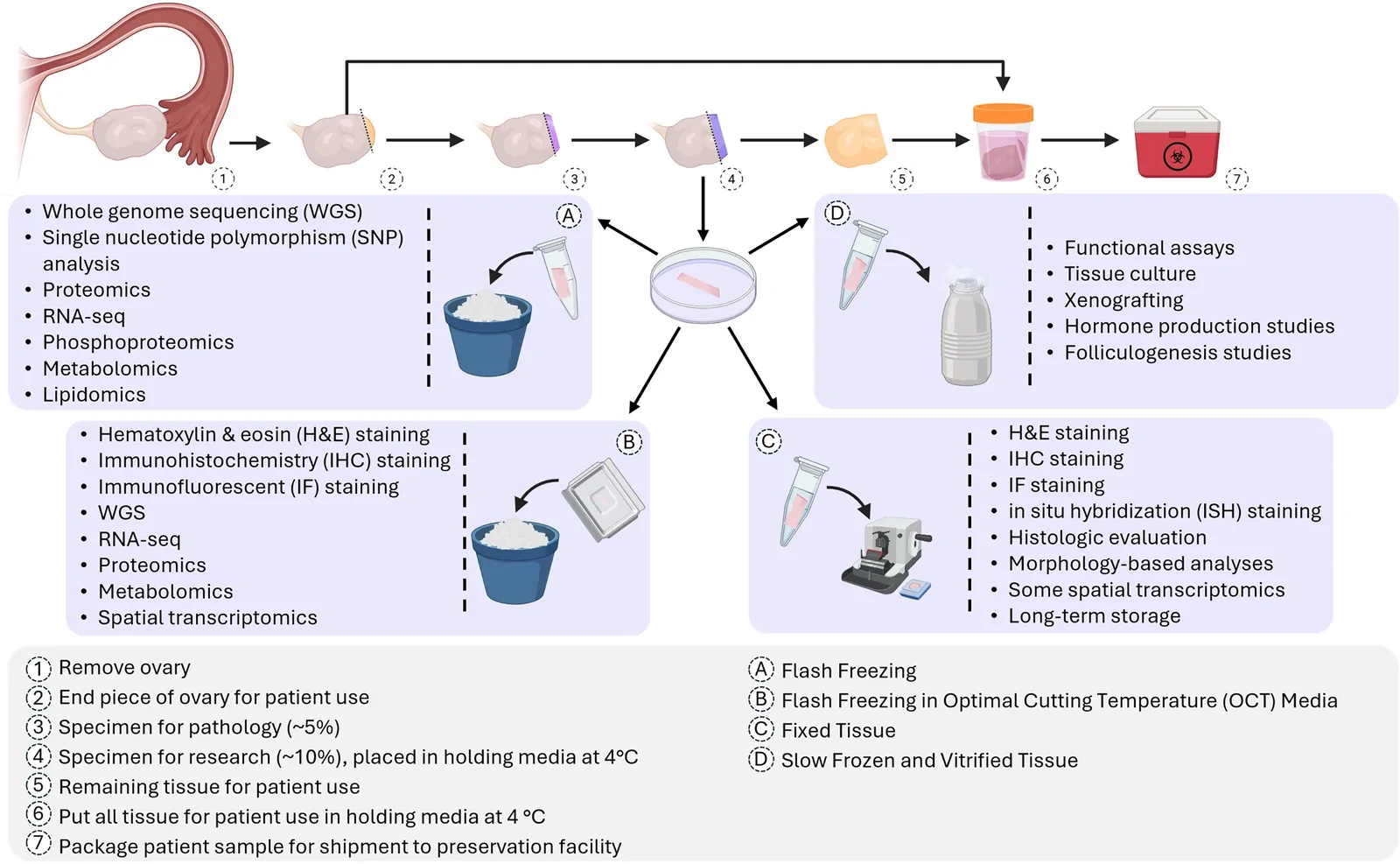
Ovarian tissue collection procedural overview.

